# Northwest Iranian dermatophyte isolates: anthropophilic and geophilic

**DOI:** 10.22034/CMM.2024.345232.1535

**Published:** 2024-05-07

**Authors:** Kambiz Diba, Kosar Jafari, Kasra Alizadeh, Narges Aslani

**Affiliations:** 1 Department of Mycology and Parasitology, School of Medicine, Urmia University of Medical Sciences, Urmia, Iran; 2 Department of Medical Microbiology and Virology, School of Medicine, Urmia University of Medical Sciences, Urmia, Iran

**Keywords:** Anthropophilic, Dermatophytosis, Epidemiology, Geophilic, Iran, *Microsporum*, *Trichophyton*

## Abstract

**Background and Purposes:**

The fungi known as dermatophytes are a group of keratinophilic agents responsible for superficial infections in humans and animals. Recognition of the species distribution and epidemiology of dermatophytosis may be helpful in the prevention and improve prophylactic measures. The present molecular epidemiology study sought to investigate the incidence of etiological agents causing dermatophytosis.

**Materials and Methods:**

The morphologic methods and polymerase chain reaction-restriction fragment length polymorphism using *MvaI* restriction enzyme were performed to identify dermatophytes isolated from the soil, compost, and clinical samples.

**Results:**

Based on findings, 39 (8.1%) clinical specimens and 10 (8.2%) environmental samples were morphologically and molecularly identified as dermatophytes. In the clinical samples, *Trichophyton mentagrophytes*/*T. interdigitale* species complex was isolated with the highest incidence rate. The dermatophytes comprise seven species of the four genera, viz., *T. interdigitale* (currently *T. mentagrophytes*, n=15, 40.5%), Microsporum canis (n=10, 27%), *T. verrucosum* (n=5, 13.5%), *T. rubrum* (n=4, 10.8%), *Myriodontium keratinophilum* (n=2, 5.4%), and *T. benhamiae* (n=1, 2.7%). The geophilic identified species included *Nannizzia gypsea* (n=5), *Arthroderma multifidum* (n=2), *Afanoascus flavisence* (n=2), and *Nannizzia fulva* (n=1).

**Conclusion:**

The current study provides a diverse overview of dermatophytes in the northwest of Iran to improve their surveillance. The present investigation of clinical specimens revealed that *Myriodontium keratinophilum*, as a species rarely detected with keratolytic properties, emerged as a causative agent of dermatophytosis.

## Introduction

The spectrum of infections caused by dermatophytes, known as dermatophytosis, Tinea, or ringworm, affects the keratinized structures, such as skin, hair, and nails. Dermatophytes are classified into nine pathogenic genera: *Trichophyton*, *Epidermophyton*, *Nannizia*, *Paraphyton*, *Lopophyton*, *Microsporum*, *Arthroderma*, *Ctenomyces*, and *Guarromycesm* [ 1 , 2 ]. Soil and compost in rural areas, skin, hair, wool, and feathers are the sources of dermatophytic infections. Based on their sources, they are classified into geophilic, zoophilic, and anthropophilic dermatophytes [ 3 ]. Frequency of dermatophytosis is variable and depends on many factors, including temperature, humidity, and host conditions, such as gender, age, and occupation [ 4 ]. Based on previous dermatophyte-associated infection studies, *Trichophyton rubrum* is the predominant causative pathogen of dermatophytosis [ 5 ], a considerable frequency of dermatophytosis is due to infection of hair and skin by the geophilic and zoophilic *Trichophyton* species. Epidemiology reports show that the prevalence of infection with rare anthropophilic dermatophytes has increased over the past decade, including *T. interdigitalis*, *T. rubrum* [ 6 , 7 ], *T. violaceum* [ 2 ] , and *T. sudanensis* [ 8 , 9 ]. However, zoophilic dermatophytes, such as *T. benhamiae*, are frequently isolated from patient samples, namely, skin, hair, and nails [ 10 ]. With this background in mind, determining the distribution of different species of dermatophytes may be helpful for provision of the health awareness in the community and the development of preventive strategies. Hence, this epidemiological study focused on isolating dermatophytes from soil, compost, and clinical samples using methods of polymerase chain reaction-restriction fragment length polymorphism (PCR-RFLP) and PCR sequencing, in different areas of West Azerbaijan province, northwestern Iran.

## Materials and Methods

### Environmental samples

For the investigation of environmental dermatophyte distribution in northwestern Iran, three geographical areas of West Azerbaijan province, including Maku (north), Miandoab (south), and Urmia (center), were studied. In total, 122 soil and compost samples were collected and transferred to the Medical Mycology Center at Urmia University of Medical Sciences from November 2019 to the end of November 2022. 

### Fungal culture

The pH of each soil sample (15 g) was measured using a digital pH meter device. Samples were then cultured for dermatophytes as follows: each sample was scattered in a sterile Petri dish containing 20 mL of sterile water, and some sterile horse or human hair strips were placed in the soil samples. The plates were incubated at room temperature for 1-10 weeks. The hair strips were removed from the plates and investigated microscopically for fungal growth. The infected hairs were transferred into the selective fungal medium (Sabouraud dextrose agar with chloramphenicol (0.05 mg mL^–1^) and cycloheximide (0.5 mg mL^-1^) and incubated at room temperature for another minimum period of one week [ 11 , 12 ].

### Clinical samples

In total, 483 clinical specimens were collected from the patients’ presenting lesions who referred to the mycology laboratory at the University Hospital in Urmia. Firstly, one part of each clinical sample was preliminarily examined using a light microscope with 15% potassium hydroxide for the invasive dermatophyte elements (septate hypha and arthroconidia). Afterward, the second portion was inoculated on Sabouraud dextrose agar medium supplemented with chloramphenicol (0.05 mg mL^–1^) plus cycloheximide (400 µg/mL). The inoculated plates were incubated at 30 °C for one to four weeks. Each grown colony was examined by macroscopic and microscopic methods for the primary identification of dermatophyte species.

### Molecular identification

Subsequently, genomic DNA was extracted from unknown isolates as previously described and stored at -80 °C before use [ 13 ]. For the identification of all strains to the species level, PCR-RFLP on the rDNA gene was performed using ITS1 and ITS4 primers (ITS-1: 5'- TCC GTA GGT GAA CCT GCG G - 3' and ITS-4: 5'- TCC TCC GCT TAT TGAT TAT GC - 3') for amplification [ 14 ] and the restriction enzyme *MvaI* for RFLP digestion [ 15 ]. Unknown isolates were identified and confirmed with ITS-rDNA sequencing. Three standard strains, namely *T. mentagrophytes* PTCC5054, E. floccosum PTCC5090, and *T. rubrum* PTCC5143 were included as the controls for molecular identification of the clinical dermatophyte isolates. 

## Results

From 483 clinical specimens, in 39 (8.1%) cases, a fungal strain was isolated which included 32 (82.1%) dermatophytes, one Candida albicans (2.6%), and one Fusarium species (2.6%) based on molecular screening (Supplementary Figure 1), and 12 (12.8%) remaining isolates with unknown RFLP patterns. Among dermatophytes, *T. mentagrophytes*/*T. interdigitale* species complex was the dominant species, followed by Microsporum canis (25.6%) and unidentified isolates (12.8%). The most commonly involved organs were nail (53.3%), foot (20%), and scalp (15.5%) (Table 1). 

**Table 1:** Clinical dermatophyte isolates identified by the conventional phenotypical methods

Clinical Site	*T. mentagrophytes*/ *T. interdigitale*	*T. verrucosum*	*T. rubrum*	Microsporum canis	*C. albicans*	Fusarium species	Unknown fungi	Total
Nail	7	-	2	1	1	1	4	16
Foot	5	1	1	-	-	-	1	8
Body	1	2	-	2	-	-	-	5
Scalp	-	1	-	6	-	-	-	7
Inguinal	1	-	-	-	-	-	-	1
Facial	-	-	-	1	-	-	-	1
Manual	-	1	-	-	-	-	-	1
Total	14 (35.9%)	5 (12.8%)	3 (7.7%)	10 (25.6%))	1 (2.6)	1 (2.6)	5 (12.8%)	39

*T; Trichophyton*, *M; Microsporum*, *C; Candida*

The unknown clinical isolates were identified by sequencing of the ITS-rDNA region, as listed in Table 2. One of the molecularly identified fungi was Myriodontium keratinophilum which was isolated from two female cases with nail infections, followed by *T. benhami* and *T. rubrum* (Table 2). Only 10 (8.2%) fungi were morphologically identified from all environmental isolates as dermatophytes. In the molecular investigation, 5 out of 10 fungi were confirmed as *Nannizzia gypsea* (formerly Microsporum gypseum), the most frequent dermatophyte species of our environmental examinations, which was isolated from three compost and two soil samples. *Arthroderma multifidum* and *Nannizzia fulva* were the dermatophytes isolates of soil and compost samples. The keratinophilic fungus, *Afanoascus flavisence*, a non-dermatophyte fungal species, was isolated from the soil (Table 3).

**Table 2:** Molecular identification of unknown clinical isolates from keratinophilic moulds

No	Accession number	Dermatophyte species	Site	Place	Gender	Age
1	PQ135858	Trichophyton benhamiae (UR-1)	Nail	Urban	Male	45
2	PQ135916	*T. mentagrophytes*/ *T. interdigitale* (UR-4)	Foot	Urban	Female	55
3	PQ135856	*Myriodontium keratinophilum* (UR-10)	Nail	Urban	Female	31
4	PQ135917	*Trichophyton rubrum* (UR-16)	Nail	Urban	Male	39
5	PQ135857	*Myriodontium keratinophilum* (UR-21)	Nail	Urban	Female	34

**Table 3:** Dermatophyte and non-dermatophyte keratinophilic fungi isolated from environmental sources

No.	Strain code	Dermatophyte species	Source	Place
1	MMR	*Arthroderma multifidum*	Soil	Urban
2	SSRS	*Afanoascus flavescens*	Soil	Stable
3	MIS	*Nannizzia gypsea*	Compost	Stable
4	MCS	*Nannizzia fulva*	Compost	Stable
5	UOSS	*Nannizzia gypsea*	Compost	Pasture
6	MIPS	*Nannizzia gypsea*	Soil	Pasture
7	USIPS	*Nannizzia gypsea*	Soil	Urban
8	STRS	*Afanoascus flavescens*	Soil	Urban
9	USIRS	*Arthroderma multifidum*	Soil	Urban
10	MLS	*Nannizzia gypsea*	Compost	Urban

## Discussion

Results of the present study showed that different dermatophytes, including *T. mentagrophytes*/*T. interdigitale*, *M. canis*, *T. verrucosum*, *T. rubrum*, *M. keratinophilum*, and *T. benhamiae*, cause clinical infections in humans in Urmia. In this study, emerging species were recovered from the clinical samples, such as *T. verrucosum*, *T. benhamiae*, and *M. keratinophilum*, compared to a previously published report in Urmia [ 16 ] . 

In similar research, Ebrahimi et al. (2019) studied the 79 dermatophyte species causing dermatophytosis in the human skin, hair, and nails in northeastern Iran. Their identified species were mainly similar to the isolates of the present study, including *T. mentagrophytes*/*T. interdigitale* as the main causative agent (46.8%), followed by Epidermophyton floccosum (15.2%), *T. rubrum* (10.1%), *M. canis* (10.1%), *T. violaceum* (6.3%), *T. tonsurans* (5.1%), *N. gypsea* (3.8%), *T. benhamiae* (1.3%), and *T. verrucosum* (1.3%). They concluded that *T. benhamiae* is an emerging agent of dermatophytosis in Mashhad, northeastern Iran [ 12 ] . Zamani et al. (2016) studied the epidemiological trends of dermatophytosis in Tehran, Iran, in a five-year retrospective survey. They reported microscopically identified infections in 2,622 (19.7%) out of 13,312 patients who were clinically suspected of cutaneous fungal infections. Tinea pedis (30.4%) was reported to be the most prevalent type of dermatophytosis, followed by tinea cruris (29.8%) and tinea corporis (15.8%). Considering the fungal species, *Epidermophyton floccosum* (31%) was found to be the most prevalent agent, followed by *T. rubrum* (26.2%) and *T. mentagrophytes*/*T. interdigitale* (20.3%). They concluded that there is a considerable distribution of dermatophytosis due to zoophilic, anthropophilic, and geophilic species among humans in varied age groups [ 17 ]. 

However, in the present study, growing diversity was found in dermatophyte species causing human infections. Moreover, the most prevalent clinical type of dermatophytosis was onychomycoses. A truly rare pathogenic agent, Myriodontium keratinophilum, was recovered in this assessment as a causative agent of onychomycoses. In this investigation, this less frequent agent was specified according to ITS sequencing. Myriodontium kertinophylum was first isolated in Italy by Samson and Polonelli [ 18 ]. Moreover, a case of frontal sinusitis isolated from the mucosal tissue lining of the sinus in a Nigerian patient was reported [ 19 ]. In addition, in the case report of Kochhar et al. from India (2018), the mentioned agent was isolated from corneal scraping and was identified based on morphological criteria [ 20 ]. 

Myriodontium keratinophilum is a fungus widespread in nature, most abundantly found in keratin-reach environments, such as feathers, nails, and hair. However, the occurrence of this rare pathogenic fungal has been known to be uncommon and it is regularly isolated from dermatophytic and psoriatic lesions infected by non-dermatophytes [ 2 , 21 ]. Furthermore, it is identified as one of the agents of hyalohyphomycosis [ 9 , 21 ]. Due to its uncertain pathogenic potential, *M. keratinophilum* is classified as safe to be handled with a bio-safety low equivalent contaminant [ 3 , 21 ]. 

Based on the ITS ribosomal DNA region sequencing, the *Trichophyton* species of *A. benhamiae* does not currently belong to the *T. mentagrophytes* complex; it is now a distinct species known as *T. benhamiae* [ 2 ]. *Trichophyton* benhamiae causes inflammatory dermatomycosis in children and adolescents. In addition to tinea capitis, it may cause tinea corporis and tinea mannum. *Trichophyton benhamiae* is a prevalent zoophilic dermatophyte, probably more frequent in some regions, compared to *M. canis*. 

Based on their macroscopic and microscopic features, the mycological identification exhibits yellow pigmentate colonies, similar to *M. canis* and *T. interdigitale* in some cases [ 8 ]. The first report of *A. benhamiae* causing tinea corporis was described by de Freitas in Brazil in 2019 [ 9 ]. In the present study, *T. benhamiae* is isolated from a clinical sample of the nail, yet at a low frequency. In a study performed by Ebrahimi et al. (2019) on the epidemiology of dermatophytosis in northeastern Iran by ITS-RFLP, *T. benhamiae* was identified as a new emerging agent that had never been recorded in Mashhad city until then. 

Cases of *T. benhamiae* affecting the hair follicles in the beard area have been reported in some European countries [ 22 , 23 ]. *Arthroderma multifidum*, as a keratinophilic fungus in soils, has been isolated from soil and rook colonies (Corvus frugilegus) and was used as a biological agent for the disposal of waste feathers. The Keratinase activity of *A. tuberculatum* and *A. multifidum* results in the release of nitrogen and sulfur in the soil and consequently is highly significant for fertilization, adding nutritionally important peptides and amino acids to the soil [ 24 ].

Investigations from Egypt and Slovakia by sequence-based and morphological surveys, respectively, found *A. multifidum*, as the spectrum of soil-inhabitant dermatophyte [ 25 , 26 ]. In the present study, *A. flavescens*, as a zoophilic dermatophyte, was isolated from two different environmental soil samples. Considering the environment, *Arthroderma multifidum*, *Afanoascus flavisence*, *N. gypsea*, and *N. fulva* are present in soil or animal-related areas. 

The dermatophytes, keratinophilic fungi, embody significant microorganisms of the soil microbiota. Some of them are cosmopolitan, and some with restricted distribution [ 27 ]. Pontes et al. (2013) studied the distribution of dermatophytes from soils of urban and rural areas of cities of Paraiba state in Brazil. Fungal growth was observed in 62% of the 212 samples they collected, especially those collected from areas with hot and humid tropical climates. Regarding soil pH, 71% of the growth occurred at pH 7.02-9.06 (alkaline). Among their isolates, 57.3% were geophilic species, particularly *T. terrestre* (31.3%) and *N. gypsea* (21.4%). They also reported *M. nanum* (*N. nana*) and *T. ajelloi* (*Arthroderma uncinatum*). Their identified zoophilic species were *T. mentagrophytes var. mentagrophytes* (31.3%) and *T. verrucosum* (7.6%), and an anthropophilic species was *T. tonsurans*. Their study showed that the soil of urban areas, including empty lots, schools, slums, and squares of cities, can be the most suitable source for almost all dermatophytes [ 27 ].

Distinguishing *M. keratinophilum* and *T. benhamiae* from clinical samples and *A. multifidum*, *A. flavescens*, *N. gypsea*, and *N. fulva* from soils and animal-related areas in West Azerbaijan province, northwestern Iran was a noticeable finding in the present study. Myriodontium keratinophilum, which was recovered in this study, has been incriminated to be a very rare pathogenic fungus in humans.

## Conclusion

In conclusion, results of the current study suggested that rare species, such as *M. keratinophilum*, that have been identified as the primary pathogens, need to be properly identified for distribution profile intention. Isolation of rare geophilic dermatophytes in the current study, including *Arthroderma multifidum* (n=2) and *Afanoascus flavisence* (n=2) was a noticeable finding. Finally, DNA-based identification for the definite identification of dermatophyte species is highly recommended.
